# Factors affecting the mesothelioma detection rate within national and international epidemiological studies: insights from Scottish linked cancer registry-mortality data

**DOI:** 10.1038/sj.bjc.6603293

**Published:** 2006-08-08

**Authors:** D R Camidge, D L Stockton, M Bain

**Affiliations:** 1 Edinburgh Cancer Centre, Western General Hospital, Crewe Road, Edinburgh EH4 2XU, UK; 2 Information Services, NHS National Services Scotland, Gyle Square, 1 South Gyle Crescent, Edinburgh EH12 9EB, UK

**Keywords:** mesothelioma, epidemiology, ICD-9, ICD-10, accidental poisoning

## Abstract

ICD-9 code 163 (malignant neoplasm of pleura) listed as underlying cause of death detected only 40% of Scottish mesothelioma cases (all body sites) from the cancer registry in 1981–1999. This is lower than both the previously published 55% figure, derived from UK mesothelioma register data 1986–1991, which is based on any mention of mesothelioma on death certificates, cross-referenced to cancer registry data, and the 44% figure derived from Scottish mortality data 1981–1999, which captured any mention of mesothelioma on the death certificate. Detection from cancer registry data increased to 75% under ICD-10 in Scotland, confirming earlier predictions of the benefit of ICD-10's more specific mesothelioma codes. Including the accidental poisoning codes E866.4 (ICD-9) and X49 (ICD-10), covering poisoning by ‘unspecified’ and ‘other’ causes, which appear to have been used as coding surrogates for mesothelioma when asbestos exposure was explicitly mentioned in deaths suggestive of a mesothelioma, and which are recorded as the underlying cause of death in 4–7% of mesotheliomas, may improve the mesothelioma detection rate in future epidemiological studies.

Mesotheliomas predominantly occur in males and in the pleura (Britton, 2002). The vast majority are caused by occupational exposure to asbestos (Peto *et al*, 1999). Incidence rates from UK cancer registries are based on histological, cytological and radiological information from a range of sources, including hospital discharge records and death certificates, the histological information extending beyond the standard International Classification of Diseases (ICD) codes. In contrast, electronic national death records, abstracted from hand-written death certificates, from which most mortality data are derived, are usually restricted to the standard ICD codes available at the time of death. ICD-9 was used in Scotland between 1979 and 1999, and ICD-10 from 1st January 2000. In part I of British death certificates, as in many other countries, a series of events are listed, the last of which should be the underlying cause of death. In part II significant conditions, diseases or events contributing to death, but not part of this sequence of events may also be listed. Despite WHO guidelines for uniformity in coding practices, for example, with regard to which of the items listed in part I or part II is chosen as the underlying cause of death, significant international variation is recognised (Percy and Dolman, 1978).

Epidemiological studies of mortality have been hampered by the lack of standardised specific codes for mesothelioma before the introduction of ICD-10. Although certain countries (including Scotland) have added supplemental codes or text fields to their death records to record any mention of mesothelioma within the hand-written certificates, this has not been uniform international practice. The majority of studies have, instead, used ICD-9 code 163 (malignant neoplasm of pleura) as a surrogate for ‘mesothelioma’ in electronic databases (Davis *et al*, 1992; Peto *et al*, 1999; Pinheiro *et al*, 2003). To assess this as a surrogate for mesothelioma, we used Scottish national data, which permits direct linkage of individual patient data from cancer registry and death records, to identify mesothelioma cases and then the codes ultimately used for underlying cause of death in these patients. In addition, by comparing the cause of death codes for cancer registry-listed mesothelioma patients dying between 1981 and 1999 and between 2000 and 2003, we explored the impact of the introduction of ICD-10 on this issue within the UK.

## METHODS

Information Services of NHS Scotland (ISD) routinely produces a data set containing SMR6 (Cancer Registration Records) and Registrar General's death records, on over 5.8 million patients, linked together using ‘probability matching.’ At the time of analysis a full data set was available from 1981 to 2002 for cancer registrations and 1981–2003 for deaths. It is estimated that the probability-matching algorithm, which uses all available identifying information (eg name, date of birth, postcode, hospital/patient reference number), links these records with an accuracy of 98% (Kendrick and Clarke, 1993). Before approximately 1997, five autonomous cancer registries within Scotland, working in slightly different ways, fed their data to ISD to generate the SMR6 database. The West of Scotland relied heavily on hospital medical record departments, supplemented by death records with any mention of cancer. In contrast the other four registries relied more heavily on histological data, supplemented by hospital records and death certificate data. Since 1997, registration has been centralised at ISD, using sources that include pathology records, hospital discharge records, death records, and notifications from private hospitals. The ICD codes used for underlying cause of death were quantified for the 2740 mesothelioma patients (all body sites) listed on the cancer registry who died between 1981 and 2003.

## RESULTS

The top 10 ICD codes used for underlying cause of death in the 2133 mesothelioma patients recorded in the cancer registry who died between 1981 and 1999 (ICD-9), and for the 607 who died between 2000 and 2003 (ICD-10) are shown in Table 1.

## DISCUSSION

A broad range of ICD-9 and ICD-10 codes have been used in codifying the underlying cause of death among mesothelioma patients in Scotland since 1981 (Table 1). Between 1981 and 1999, using ICD-9, only 861 of 2133 (40%) deaths among mesothelioma cases were encoded with malignant pleural tumours as the underlying cause (3 × 163.0, 1 × 163.1, 2 × 163.8, 855 × 163.9), the code category most commonly used for mesothelioma epidemiological research. Beyond pleural mesotheliomas that may be being missed, the use of several other body site codes for malignant neoplasm within the cancer registry deaths is also apparent (eg peritoneum 2% or unspecified site 19%), potentially reflecting the occurrence of the less common forms of mesothelioma, outwith the pleura, which are not being picked up by the 163-based codes. The under-reporting of mesotheliomas from ICD-8/9 based death certificate pleural tumour codes alone is well recognised (Davis *et al*, 1992; Iwatsubo *et al*, 2002). However, within the UK, it has not caused particular problems due to the existence of a specific high-quality UK mesothelioma register (England, Wales and Scotland) that captures any mention of the term ‘mesothelioma’ from the hand-written death certificates, in addition to cross-referencing data derived from cancer registry records (McElvenny *et al*, 2005). The key significance of knowing both death certificate data and cancer/mesothelioma registry data within the same country, relates to the interpretation of reported mesothelioma-specific death rates, and trends in death rates, between countries. The detection rate is derived from the percentage of ‘genuine’ mesotheliomas whose deaths are encoded as 163. Internationally, different sources of the denominator (the ‘genuine’ number) in published detection rates have been used, including cases followed-up from cancer registries in the USA (Davis *et al*, 1992), specialist mesothelioma registries in Italy and the UK (Peto *et al*, 1999; Gorini *et al*, 2002), and histological series in France (Iwatsubo *et al*, 2002). The confirmation rate is derived from the percentage of 163 coded deaths that appear related to ‘genuine’ mesotheliomas, according to whether they are also entered as mesotheliomas within cancer or mesothelioma registries (Davis *et al*, 1992; Peto *et al*, 1999), or through case-by-case explorations of medical notes/pathology (Iwatsubo *et al*, 2002; Pinheiro *et al*, 2003). By employing compensatory calculations based on, for example, the ratio of mesothelioma to pleural cancer mortality (confirmation rate divided by detection rate), 163-encoded death rates between countries can be more informatively compared (Peto *et al*, 1999; Gorini *et al*, 2002).

The 40% detection rate we report, based on Scottish cancer registry data 1981–1999, is lower than the 55% figure, based on UK mesothelioma registry data 1986–1991, used by Peto *et al* (1999) when assessing trends in mesothelioma mortality rates across Europe. Although some temporal variation in detection rates is recognised (Peto *et al*, 1999), the different sources of ‘genuine’ mesotheliomas used (mesothelioma registry *vs* cancer registry) may also be important. The detection rate comparison was still 40 *vs* 44%, when 1981–1999 Scottish cancer-registry data and Scottish mortality data, capturing any text mention of mesothelioma on the death certificate by supplemental code (feeding into the UK mesothelioma register) were used, respectively. Although the overall pattern was similar, further subtle differences in absolute numbers and percentages in the top ten ICD-9 codes used for underlying cause of death were apparent when these two different sources of mesothelioma denominator information were used, despite them being derived from the same basic population (Table 2). Consequently, there may need to be additional interpretation of the compensatory calculations used between countries with different sources for their definitive incidence numbers. With regard to the specificity of a 163 code, between 1981 and 1999, 83% (829/997) of Scottish deaths coded 163 in the underlying cause field were registered as mesotheliomas on the cancer registry, in line with the UK confirmation rate figure of 89% used by Peto *et al*.

Only codes used for the underlying cause of death were addressed in this study. Previous studies support this approach, with the inclusion of data outwith the underlying cause of death field making little (Davis *et al*, 1992) or no difference (Iwatsubo *et al*, 2002) to published mesothelioma detection rates. Partly this may be due to the fact that the most appropriate underlying cause in the final encoded data is selected from any field on the death certificate, either by human or automated software abstraction, and as mesotheliomas have such a poor prognosis, they may frequently be chosen as the underlying cause whatever field they are entered in on the death certificate. To illustrate this, in the 2133 Scottish mesothelioma cases who died between 1981 and 1999, although 351 had 163 codes in positions other than underlying cause of death (16%), 289 of these also had 163 listed under the underlying cause.

Between 2000 and 2003, following the introduction of ICD-10, 75% (455/607) of Scottish mesothelioma deaths using cancer registry data could be accounted for by the new mesothelioma-specific C45 codes. This is in line with earlier USA predictions (80%), and preliminary estimates (82%) based on crude state-specific population mortality/incidence ratios, made without the benefit of direct record linkage data (Davis *et al*, 1992; Pinheiro *et al*, 2004). Most of the increased detection in the Scottish data set appears to result from a drop in the use of codes relating to ‘malignant neoplasm of bronchus and lung’ and ‘malignant neoplasm, unspecified site’ from 22 to 4%, and 18 to 1%, in ICD-9 and ICD-10, respectively. Although a certain number of mesotheliomas listed on death certificates will still be being missed, through not being identified as the underlying cause of death, it should be borne in mind that a proportion of the remaining non-C45 (and non-163 codes under ICD-9) codes will always be accurate, as not all mesothelioma patients will die from their mesotheliomas. With regard to the specificity of a C45 death code, between 2000 and 2002, 95% (379/399) of ICD-10 C45 deaths were registered as mesotheliomas on the cancer registry.

The proportion of mesothelioma deaths attributable to ischaemic heart disease as the underlying cause appears constant between ICD-9 and ICD-10 at 2–3%. Similarly, the proportion of deaths attributed to specific accidental poisoning codes (E866.4 in ICD-9 and X49 in ICD-10) appears fairly constant between 4 and 7%. Although there is some evidence that the risk of accidental and deliberate self-harm may be increased among those diagnosed with cancer (Yousaf *et al*, 2005), the General Register Office for Scotland reports that these specific codes, covering poisoning by ‘unspecified’ or ‘other’ causes, are primarily being used as a surrogate for mesothelioma whenever explicit mention of asbestos exposure was apparent in the appropriate context of death suggestive of a mesothelioma (of note, asbestosis *per se* has different codes). If such coding patterns are followed internationally and use of these codes is not extensively employed for other conditions – in Scotland 81% (79/97) of underlying cause of deaths encoded E8664, 1981–1999 and 65% (36/55) of deaths encoded X49, 2000–2003 were listed as mesotheliomas in the cancer registry – 86% (83/97) and 69% (47/68), respectively based on Scottish mortality data with supplemental mesothelioma code use – they may represent an additional way to identify mesothelioma deaths, beyond ICD-9 code 163 and ICD-10 C45, within epidemiological studies in the future.

## Figures and Tables

**Table 1:** Top 10 ICD-9 and ICD-10 codes used for underlying cause of death among Scottish mesothelioma patients (cancer registry data), 1981–2003

**Total mesothelioma deaths 1981–1999=2133**			**Total mesothelioma deaths 2000–2003=607**		
**ICD-9**	**Definition**	**Observed (%)**	**ICD-10**	**Definition**	**Observed (%)**
163	Malignant neoplasm of pleura	861 (40%)	C45.0	Mesothelioma of pleura	311 (51%)
162	Malignant neoplasm of bronchus and lung	475 (22%)	C45.9	Mesothelioma, unspecified	75 (12%)
199	Malignant neoplasm, unspecified site	399 (19%)	C45.1–45.8	Mesothelioma of other sites	69 (11%)
E866.4	Accidental poisoning by other metals and their compounds and fumes	79 (4%)	X49	Accidental poisoning/exposure to other & unspecified chemicals & noxious substances	45 (7%)
158	Malignant neoplasm of peritoneum	52 (2%)	C34	Malignant neoplasm of bronchus or lung	23 (4%)
501	Asbestosis	45 (2%)	C38.4	Malignant neoplasm of pleura	17 (1%)
195.1	Malignant neoplasm of thorax	41 (2%)	C76.1	Malignant neoplasm of thorax	9 (1%)
410	Acute myocardial infarction	37 (2%)	I21	Acute myocardial infarction	7 (1%)
414	Chronic ischaemic heart disease	18 (1%)	I25	Chronic ischaemic heart disease	6 (1%)
485	Bronchopneumonia	14 (1%)	C80X	Malignant neoplasm, unspecified site	6 (1%)

**Table 2:** Top 10 ICD-9 codes used for underlying cause of death among Scottish mesothelioma patients: cancer registry data versus Scottish mortality data with supplemental mesothelioma code use, 1981–1999

**Total mesothelioma deaths (cancer registry) 1981**–**1999=2133**			**Total mesothelioma deaths (supplemental mesothelioma code use) 1981**–**1999=2002**		
**ICD-9**	**Definition**	**Observed (%)**	**ICD-9**	**Definition**	**Observed (%)**
163	Malignant neoplasm of pleura	861 (40%)	163	Malignant neoplasm of pleura	882 (44%)
162	Malignant neoplasm of bronchus and lung	475 (22%)	199	Malignant neoplasm, unspecified site	402 (20%)
199	Malignant neoplasm, unspecified site	399 (19%)	162	Malignant neoplasm of bronchus and lung	377 (19%)
E866.4	Accidental poisoning by other metals and their compounds and fumes	79 (4%)	E866.4	Accidental poisoning by other metals and their compounds and fumes	83 (4%)
158	Malignant neoplasm of peritoneum	52 (2%)	501	Asbestosis	60 (3%)
501	Asbestosis	45 (2%)	158	Malignant neoplasm of peritoneum	53 (3%)
195.1	Malignant neoplasm of thorax	41 (2%)	195.1	Malignant neoplasm of thorax	32 (1.6%)
410	Acute myocardial infarction	37 (2%)	410	Acute myocardial infarction	27 (1%)
414	Chronic ischaemic heart disease	18 (1%)	414	Chronic ischaemic heart disease	15 (1%)
485	Bronchopneumonia	14 (1%)	195.2	Malignant neoplasm of abdomen	7 (<1%)

*Source:* General Register Office for Scotland.
